# Video data recovery from slack space of pre‐allocated video files

**DOI:** 10.1111/1556-4029.70412

**Published:** 2026-07-16

**Authors:** Junseok Byun, Kyu‐Sun Shim, Yongseok Kim

**Affiliations:** ^1^ Department of Computer Science and Engineering Kangwon National University Chuncheon‐si Gangwon‐do Republic of Korea; ^2^ Digital Analysis Division National Forensic Service Wonju‐si Gangwon‐do Republic of Korea

**Keywords:** dashboard camera, digital forensics, file carving, file slack, frame‐based recovery, video recovery

## Abstract

The recovery of video evidence from dashboard cameras is often compromised by damage, file system errors, or circular recording mechanisms that overwrite older data. Traditional digital forensic techniques primarily focus on file carving and frame recovery from the unallocated area of storage media. However, this approach is often insufficient for dashcams that use pre‐allocation strategies, where fixed‐size files are created before recording. As a result, residual data from previous recordings may remain in unused portions of these allocated files but are typically overlooked during forensic investigations. In this study, we identify and define a forensic artifact termed “video file slack,” which exists within the allocated space of pre‐generated files and contains valid video data from previous recording cycles. To our knowledge, this study is among the first to investigate video recovery from slack regions created by dashcam pre‐allocation mechanisms. We propose a method to extract the slack area from common container formats (AVI, MP4, and ASF) and reconstruct fragmented H.264 video streams using frame‐based recovery techniques. Experimental results show that the proposed method expands the recoverable area by up to 966.7% compared with traditional analysis limited to the unallocated area. A hit‐and‐run case study further demonstrates that frames capturing the moment of impact can be recovered from video file slack even when no data are recovered from the unallocated area. These findings indicate that Video File Slack is a valuable source of forensic evidence in dashcam storage systems and that slack‐based recovery can improve the completeness of digital forensic investigations.

**Highlights**

- “Video File Slack” is defined as a novel evidentiary repository in dashcam investigations.
- A novel method is proposed to extract residual video data from pre‐allocated file structures.
- Slack‐based recovery is proven to retrieve critical evidence when unallocated space yields nothing.

## 1 INTRODUCTION

The significance of video evidence in digital forensics is rapidly increasing. Video data are increasingly leveraged as pivotal evidence in legal proceedings because of their unique capacity to provide a vivid and objective reconstruction of events at a crime scene [1, 2]. In particular, video from vehicle dashboard cameras (dashcams) serves as objective evidence in legal proceedings, providing a clear record of traffic accidents and criminal activities [3, 4, 5]. However, the recovery of this critical evidence is frequently compromised. Factors such as physical damage to the storage device, file system errors, and the inherent circular recording (loop recording) function often lead to the deletion or overwriting of crucial video files. Furthermore, sudden shocks during an accident can prevent the video from being saved correctly. While dashcam video recovery can be instrumental in reconstructing a crime scene, the likelihood of successful restoration diminishes significantly when the device is secured long after an incident, primarily because of circular recording or intentional deletion.

To address this challenge, much previous research has proposed file carving techniques targeting the unallocated area, operating at the file level. However, conventional file‐level carving is often unsuitable for video recovery because the large size of video files makes it difficult to restore them in their entirety, especially in fragmented environments. In this constrained context, frame‐by‐frame (or frame‐based) recovery techniques were introduced, significantly improving recovery rates [6, 7, 8]. Despite this advancement, existing research remains limited, with an almost exclusive focus on the unallocated area.

Recently, dashcam recording storage mechanisms have transitioned to utilizing partition cluster allocation and pre‐allocation techniques [9]. By pre‐allocating clusters upon file creation, these techniques minimize both the frequency of metadata updates during recording and the number of overwrite operations on the memory storage. This shift is primarily driven by the inherent vulnerabilities of FAT‐based file systems, which are typically employed in dashcam storage cards. Specifically, the FAT file system generates frequent entry updates to the FAT table and directory during continuous recording, rendering it highly susceptible to corruption in the event of sudden power loss or memory errors. Furthermore, the circular recording mechanisms commonly implemented in dashcams aggravate severe file fragmentation, which in turn induces frequent random write operations that degrade the performance of the storage card and shorten its lifespan. To mitigate these issues, the pre‐allocation strategy establishes a maximum file size within the system configuration. During the initial formatting of the storage card, the system pre‐allocates clusters corresponding to this fixed maximum size for the video files, thereby allowing the system to operate more reliably by reducing runtime allocation overhead. In this study, we focus on the observation that when the actual video size is smaller than the pre‐allocated file size, portions of previously recorded video data may remain after the end of the current recording. Notably, from the perspective of the file system, this residual data is not classified as unallocated space, making it a unique area of interest for recovery.

Herein, we propose a recovery technique specifically adapted to the unique storage characteristics of dashcams. Rather than focusing on traditional file system slack, which typically refers to the difference between a file's logical size and its allocated physical size, our approach targets the substantial slack area generated by the pre‐allocation mechanism. Specifically, we focus on recovering the residual data located in the significant gap between the actual recorded video size and the pre‐allocated fixed size. As illustrated in Figure 1, dashcams often pre‐allocate fixed‐size blocks for video files, resulting in a substantially larger slack area than is typically found in conventional file systems, namely the space between the end of the logical file and the end of the last cluster. For example, as shown in Figure 1, all video files recorded on the test dashboard camera memory cards are identically allocated at 62,914,560 bytes, and the video stream is subsequently written into this space. Consequently, the standard file system slack is nonexistent (0 bytes) in Figure 1 because the file is pre‐allocated irrespective of the actual content size. However, because the “Video File Slack” introduced in this paper is derived by subtracting the actual video stream size from the total physical file size, it results in a substantial area greater than 0 bytes. Based on this observation, this research investigates a novel video recovery method that specifically targets the file slack unique to pre‐allocated dashcam video files.

**FIGURE 1:**
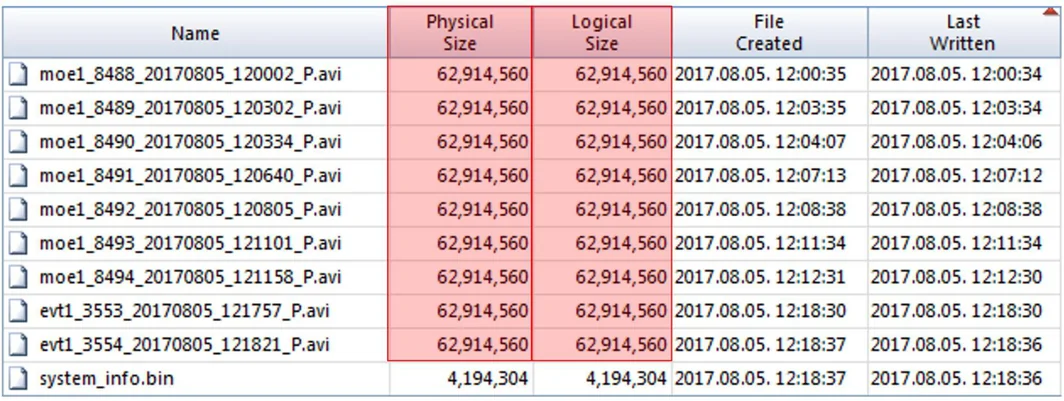
Example of pre‐allocated video files on dashcam storage.

In this study, we first propose a method to systematically collect these specific slack areas from allocated video files (e.g., MP4). Subsequently, continuous video frames within the slack data are clustered by leveraging metadata such as time information, forming “Relatively Continuous” segments. The video is then reconstructed from these segments using a frame‐by‐frame recovery technique.

Through experimentation, we demonstrate the necessity of this approach by comparing the volume of the unallocated area with the additionally collected video file slack, thereby quantifying the substantial increase in the total recoverable data target and the corresponding recovery rate. Furthermore, the practical efficacy of the proposed method is validated through a case study in which footage of a hit‐and‐run accident is successfully recovered from a video file slack.

The main contributions of this study can be summarized as follows:

- We identified and verified the unique characteristics of file slack in video files stored using pre‐allocation methods specific to dashcams, thereby expanding the total area available for video recovery.
- We developed a novel methodology to systematically collect video file slack and reconstruct relatively continuous segments on a frame‐by‐frame basis, enabling the recovery of additional video evidence.
- We demonstrated the practical importance and effectiveness of video recovery from file slack through a real‐world case study.

The remainder of this paper is organized as follows. Section 2 reviews related work and existing literature relevant to this study. Section 3 details the proposed methodology, explaining the technique for extracting “Video File Slack” based on specific file characteristics and the process for recovering video frames from this area. Section 4 validates the effectiveness of the proposed method through experiments and case studies, demonstrating the extent of video data recovery. Finally, Section 5 concludes the paper with a summary of the findings.

## 2 RELATED WORK

Several studies in digital forensics have addressed the recovery of damaged or deleted video files, demonstrating continuous advancement in the field. In particular, prior research has focused on file carving techniques and frame‐level video recovery within the unallocated area [6, 7, 8, 10, 11, 12]. Two research directions in video recovery can be identified. The first is the traditional approach centered on unallocated space, which has long been the main target of file carving techniques [13]. The second involves frame‐based recovery techniques that leverage intrinsic characteristics of multimedia files, shifting the unit of recovery from the file to the frame [4, 6].

### 2.1 File carving techniques and unallocated space centric research

File carving is a fundamental forensic technique used to recover data directly from the raw data of a storage medium based on unique file signatures or internal structures when file system metadata is corrupted or unavailable. The primary target for these carving techniques has traditionally been the “unallocated area,” defined as the space on a storage device that is not currently referenced by the file system [13, 14]. This area has attracted significant attention because it has a high probability of containing deleted file contents in relatively large, contiguous blocks, making it the most suitable target for file‐level recovery attempts.

Early file carving techniques relied on simple searches for file header and footer signatures [11, 15, 16, 17]. Although this approach is fast and straightforward, it has a significant limitation: it fails when encountering file fragmentation, a phenomenon in which files are stored across non‐contiguous clusters. On storage media such as dashcam storage, which undergo frequent file creation and deletion cycles, file fragmentation occurs with very high probability [18].

To address the problem of fragmentation, advanced techniques have been proposed, including structure‐based carving, which analyzes a file's internal structure, and content‐based carving, which utilizes the statistical properties of data [16]. Despite these technological advancements, the research focus has remained centered on the unallocated area. Indeed, a recent study analyzing the proprietary file systems of in‐vehicle digital video recorders (DVRs) reported the successful recovery of additional video evidence by extracting data from the unallocated area and restoring it on a frame‐by‐frame basis.

Traditional file carving techniques are limited to recovering data from unallocated space and function effectively only when video headers and footers remain intact. Consequently, file slack has typically been excluded from the scope of recovery targets. To address this limitation, this study proposes a frame‐based video recovery method specifically designed for “Video File Slack,” which also encompasses the traditional file slack area.

### 2.2 Advancements in frame based approaches for video recovery

The complex structure of multimedia files, particularly video, presents a significant challenge to traditional file carving methods. Video container formats such as MP4 do not rely on sample header and footer structures, and their internal data streams are highly compressed and structured. To overcome these issues, an innovative approach emerged that focuses on the fundamental unit of the video streams, the “frame,” rather than the file.

The frame‐based recovery technique proposed by Na et al. actively leverages the structural properties of the MPEG4‐Visual and H.264 video stream [6]. Both the MPEG‐4 Visual and the H.264 Start Code (Annex B) formats employ a similar mechanism for identifying and extracting frame data. In these formats, video data are organized as sequences of Network Abstraction Layer (NAL) units that are preceded by unique start codes (e.g., 0x000001). Because each NAL unit begins with such a start code, scanning for this byte pattern enables detection of frame boundaries within the bitstream. Consequently, even when the beginning of the file is missing, the presence of these start codes allows the system to identify individual frames or frame slices. The method proposed by Na et al. [6] performs a sequential scan to detect start codes, extracts candidate frame segments, and forwards them to a video decoder for validation. Successfully decoded frames are then reassembled to reconstruct the video stream.

In addition, the H.264 Length Type format (AVCC) does not rely on fixed start codes. Instead, each NAL unit is preceded by explicit length information that specifies the size of the subsequent data segment. Frame boundaries can therefore be determined by parsing the length fields recorded before each NAL unit. In this structure, parameter sets (sequence parameter set and picture parameter set) are preceded by 2‐byte length fields, whereas frame data are preceded by 4‐byte length fields. By reading these length values sequentially, the exact size and boundaries of each NAL unit can be extracted. The authors proposed a frame‐level recovery method that reconstructs candidate frames from these extracted units and verifies their correctness using an H.264 decoder.

This frame‐based recovery strategy relies on the hierarchical encoding structure of video streams, which consist of intra‐coded frames (I‐frames, or IDR frames in H.264) and inter‐coded frames (P‐frames and B‐frames in H.264). Intra‐frames represent complete images that can be decoded independently without referencing other frames, thereby serving as fundamental reference points for video reconstruction. By analyzing start codes or NAL unit headers within the bitstream, intra‐frames can be reliably identified. This property enables robust frame‐level recovery and reconstruction of damaged or fragmented video data.

Thus, frame‐based recovery technology represents a significant advancement in the video forensics field and has been demonstrated as a powerful methodology for recovering video fragments, even in the absence of metadata [4]. However, as previously noted, this robust technique has been applied almost exclusively to the unallocated area. This research diverges at this critical juncture by applying the validated framework of frame‐based recovery to file slack, a domain that has been largely overlooked.

Traditional frame‐based video recovery techniques remain primarily concentrated on the unallocated area while largely neglecting file slack regions within allocated files. The limited consideration of “Video File Slack,” which is extractable via specific dashcam pre‐allocation features, suggests that a substantial amount of recoverable data may be unintentionally excluded from the recovery process.

To address this limitation, the objective of this study is not to develop a completely new video carving algorithm, but rather to clearly define the concept of “Video File Slack” and propose a systematic method for its extraction. Once this specific slack area is successfully identified and extracted, the subsequent process of recovering video frames from the residual data involves the application of the same frame‐based recovery techniques established in previous studies. By shifting the target of extraction from unallocated space to this newly defined Video File Slack, our approach effectively recovers critical evidence that traditional paradigms overlook.

## 3 PROPOSED METHOD

In this study, we propose a video recovery technique targeting Video File Slack, a forensic artifact identified within dashboard camera video files, as described in Section 1. The proposed technique enables the recovery of substantially more video data from dashcam video file slack, an area that has been largely overlooked in conventional forensic analysis. The methodology is structured into two primary phases: (1) a slack extraction phase, which systematically collects this specific file slack from allocated video files; and (2) a frame recovery and reconstruction phase, which restores individual video frames and identifies “Relatively Continuous” segments for reassembly.

### 3.1 Video file slack extraction

In general, file slack refers to the residual space between the logical end of a file and the end of the last cluster allocated to it, arising from files being written in cluster‐based units [16, 17, 18, 19]. The size of conventional file slack is therefore inherently limited to a maximum of cluster size minus 1 byte, and recoverable data within this space is often restricted to small fragments such as partial log or text information. However, as shown in Figure 1, the dashcam video files analyzed in this study employ a different storage mechanism. A fixed file size, such as 62,914,560 bytes, is pre‐allocated by the file system for both logical and physical clusters before video data is written. Consequently, the resulting video file slack is substantially larger than the conventional file slack area found in standard file systems.

The video file slack recorded on the dashboard camera memory card, as defined in this study, refers to the unused area remaining at the end of a file after the video file is created in the file system, as shown in Figure 2A. Another type of slack area occurs when previously recorded data remains behind the region where audio and video data are stored, as illustrated in Figure 2B. This situation arises when the start position of the index is fixed while the video length is variable. Because the length of audio and video data varies according to the compression codec, sufficient space must be reserved to ensure reliable recording when designing the structure of a video file. This design choice is often combined with a circular recording process. After video files have been created up to the storage medium capacity, the FAT entry for the oldest file is deleted, and video recording continues at that location. At this point, previously recorded data remains in the slack area that is inevitably generated by the video file structure.

**FIGURE 2:**
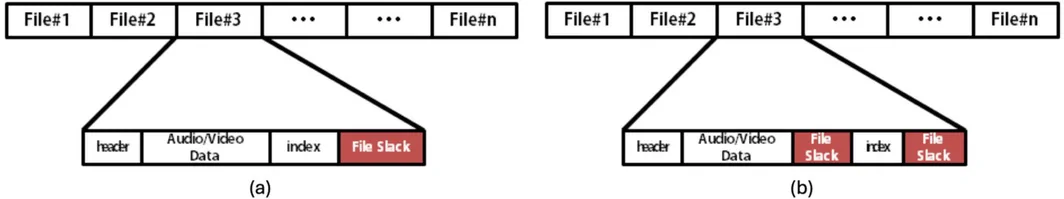
Slack area of a dashcam video file: (A) Type 1 and (B) Type 2.

#### 3.1.1 Slack extraction from the AVI format file

As illustrated in Figure 1, all video files recorded on the test dashboard camera memory cards exhibit an identical pre‐allocated size of 62,914,560 bytes. Analysis confirms that these files adhere to the Resource Interchange File Format (RIFF) structure specific to the AVI format.

The RIFF structure, depicted in Figure 3, is primarily composed of three components: a header (e.g., “hdrl”), the main audio and video data payload (e.g., “movi”), and an index chunk (e.g., “idx1”) [20]. A key observation is that previously recorded video streams persist in the residual space following the “idx1” index.

**FIGURE 3:**
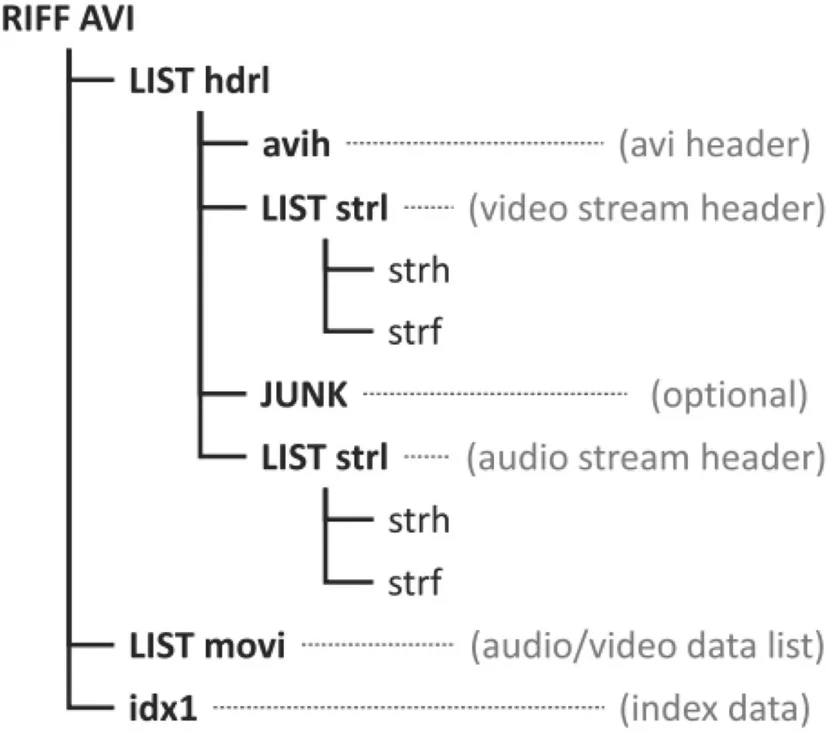
AVI video file structure.

In the AVI file structure, the total logical file size is specified as a 4‐byte little‐endian value located at the offset immediately following the “RIFF” identifier (offset 4). This logical size marker serves as the key element in the extraction process. As depicted in Figure 4, video file slack is isolated and extracted by reading all data from the specified logical end‐of‐file offset to the physical end of the pre‐allocated file.

**FIGURE 4:**
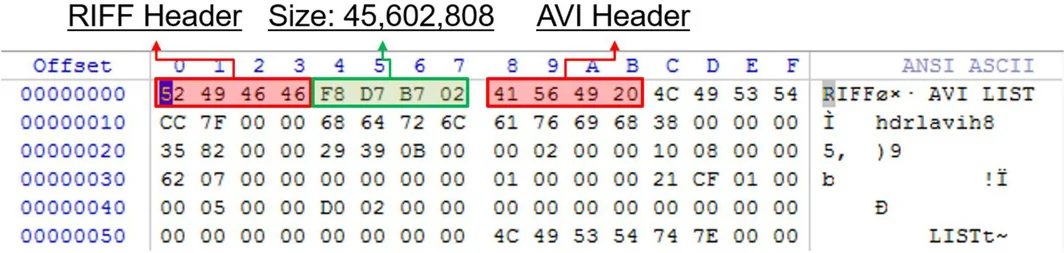
File header of the AVI video format file.

Upon extracting the raw data stream from the corresponding slack area and processing it using the codec specified in the AVI header, coherent video frames are successfully recovered from the extracted slack data, confirming that viable video data resides in this previously overlooked space.

#### 3.1.2 Slack extraction from the MP4 format file

As illustrated in Figure 5, an MP4 format file typically begins with an “ftyp” (file type) atom, and the video data payload is stored within the “mdat” (media data) atom [21]. The total length of this video data is specified by a 4‐byte field following the “mdat” identifier string, which is typically recorded in big‐endian byte order.

**FIGURE 5:**
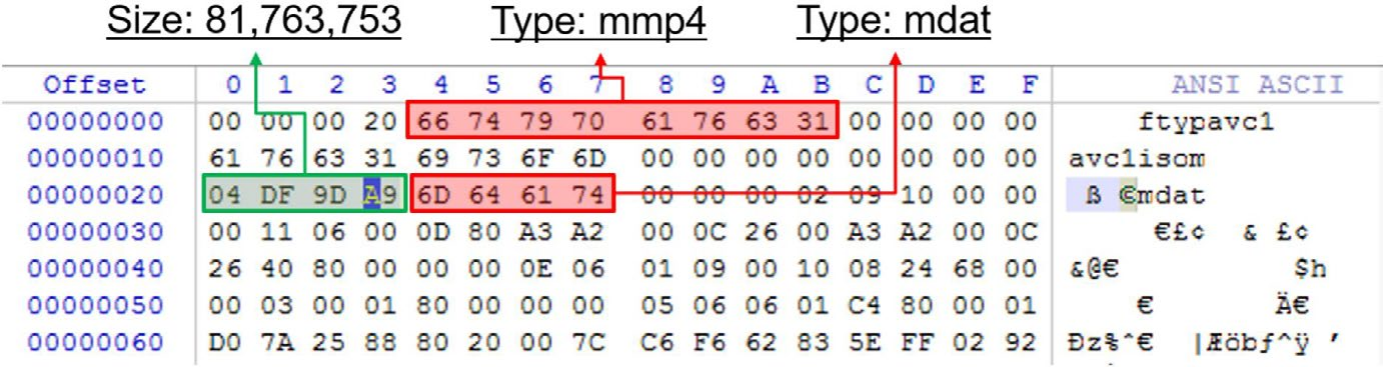
File header of the MP4 video format file.

However, implementation inconsistencies may occur. In some cases, this length field is written in little‐endian format, whereas in other instances, the metadata specifying the total length may be entirely absent, leaving the data size undefined. Nonetheless, in the general case, the logical end position of the corresponding video data can be determined by parsing this 4‐byte length field. The region extending from this calculated position to the physical end of the pre‐allocated file is therefore defined as the video file slack area.

#### 3.1.3 Slack extraction from the ASF (WMV) format file

As depicted in Figure 6, files using the Advanced Systems Format (ASF) provide another representative example. These files begin with a 128‐bit Globally Unique Identifier (GUID), and the total logical file size is specified by a 64‐bit (8‐byte) little‐endian value located immediately after this header GUID [22]. This metadata field enables precise determination of the logical end of the file data. Consequently, the region extending from this specified logical end‐of‐file offset to the physical end of the pre‐allocated file is defined as the video file slack area.

**FIGURE 6:**
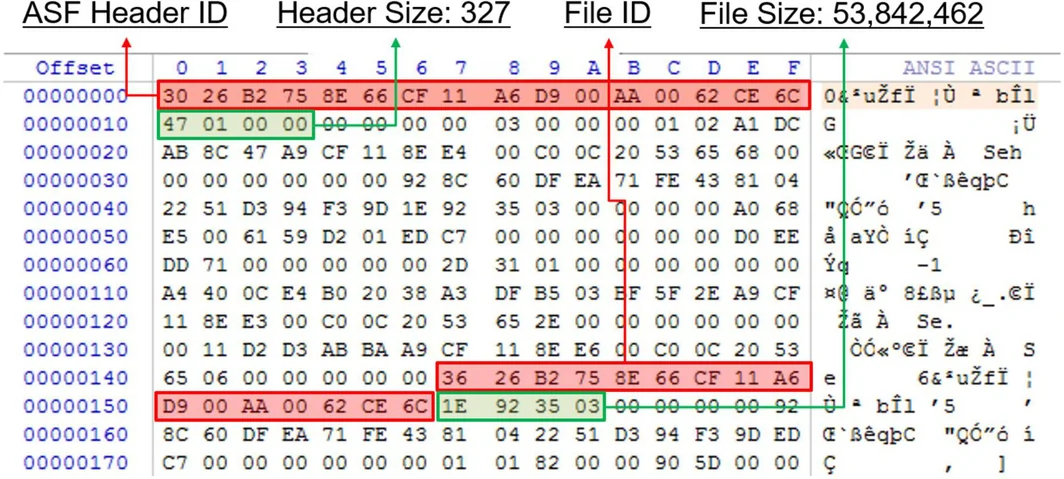
File header of the ASF video format file.

Furthermore, these principles can be extended to less structured or proprietary formats. In cases where explicit video data length information is absent, or when a non‐standard manufacturer‐proprietary format is used, residual data from previous recordings may remain in unused areas following a specific identifiable string or hexadecimal signature. This region also constitutes a form of video file slack. In addition, as illustrated by slack area type 2 in Figure 2B, the space between the logical end of the audio and video data payload and the start of a subsequent index block is also defined and targeted as a recoverable slack area in the proposed methodology.

### 3.2 Video frame recovery in video file slack

The extracted video file slack area may contain residual fragments of previously stored video data, which can potentially be recovered using a frame‐based recovery approach. This is because data located in the video file slack area are stored without preserving the original logical file structure. Consequently, conventional file‐based recovery techniques are insufficient for recovering meaningful video data. Therefore, recovering video data from slack space requires the identification and reconstruction of frame‐level data based on the structural characteristics of the video codec. In this study, the frame‐based recovery method proposed in previous work [6] was adopted to analyze residual video data in slack space. Based on this approach, video frame recovery was performed for the H.264 codec, which is widely used in contemporary dashcam systems.

An H.264 video stream stores video data in units of NAL units, each of which contains different types of information required for decoding. Successful decoding requires the presence of essential parameter sets, viz., the SPS and PPS, as well as slice data, which contains the actual compressed video frames. The SPS and PPS provide critical information required by the decoder to interpret the video stream, including the video profile, level, resolution, and entropy‐coding mode. Slice data contains the encoded image information that forms the visual content of the video. Furthermore, H.264 video streams consist of different frame types, including I‐frames (IDR frames), P‐frames, and B‐frames. Among these, the IDR frame plays a crucial role in video reconstruction because it can be decoded independently without referencing other frames.

H.264 video data can be represented in two different storage formats, the Start Code (Annex B) format and the Length Type (AVCC) format. Although both formats use the same codec, they differ in how the boundaries of NAL units are represented. The H.264 Start Code (Annex B) format, defined in the ISO/IEC 14496‐10 standard, indicates the beginning of each NAL unit using a start code, either 0x00000001 or 0x000001. This start code serves as a delimiter for identifying NAL unit boundaries. The type of NAL unit can be determined by analyzing the lower five bits of the byte immediately following the start code. For example, a value of 7 (e.g., 0x67) indicates an SPS, while 8 (e.g., 0x68) represents a PPS. A value of 5 (e.g., 0x65) corresponds to an IDR frame, which can be decoded independently without referencing other frames, whereas a value of 1 indicates a non‐IDR frame, typically corresponding to P‐ or B‐frames that require reference to previously decoded frames. Due to these structural characteristics, NAL unit boundaries can be identified by scanning for specific byte patterns, allowing SPS, PPS, and slice data to be extracted from the slack region.

In contrast, the H.264 Length Type (AVCC) format does not use start codes. Instead, each NAL unit is preceded by a length field that specifies the size of the following data. Importantly, in AVCC implementations, SPS and PPS NAL units are structurally separated from the slice data representing I‐, P‐, and B‐frames. They are typically stored in the file container metadata, such as the “avcC” box within the “stsd” (sample description) box of the “stbl” (sample table). Because the boundaries of NAL units are determined by length information rather than fixed byte patterns, the length field must first be interpreted to determine the size and boundary of each NAL unit during data analysis. Owing to this architectural design, SPS and PPS are generally absent from the continuous slice‐data stream recovered from video file slack. Therefore, during recovery from slack space, the length values are first analyzed to calculate the range of each NAL unit, enabling the identification of SPS, PPS, and slice data. Consequently, parameter information may need to be reconstructed using a reference video file, particularly when the metadata containers are partially overwritten or missing.

In this study, the data extracted from slack space were analyzed based on the structural characteristics described above, following the approach proposed in [6]. NAL units were identified, and the necessary SPS, PPS, and slice data were combined to reconstruct frame‐level video data. The reconstructed video stream was then validated using an H.264 decoder to verify both the integrity of the recovered data and the feasibility of actual decoding. If the SPS and PPS from the original file were overwritten or missing, the parameter information was extracted from a reference video file encoded with the same codec, resolution, and encoding options and used during the reconstruction process. Through this method, fragmented video data remaining in slack space could be reconstructed at the frame level and verified as playable video frames.

### 3.3 Video recovery and reconstruction

When video file slack areas extracted using the proposed method are decoded with existing techniques such as frame‐based recovery, embedded metadata including date and time information is often rendered as a visual overlay on the recovered video data, as illustrated in Figure 7. As proposed by Shim et al. [23], Optical Character Recognition (OCR) can be applied to extract the timestamp information displayed in each video frame. This information is critical for determining whether the recovered data is relevant to the incident under investigation. However, timestamps extracted through OCR may not always be perfectly accurate. In such cases, validation of the recovered frames becomes challenging and may require manual visual inspection of potentially thousands or even tens of thousands of individual frames, which introduces a significant practical limitation.

**FIGURE 7:**
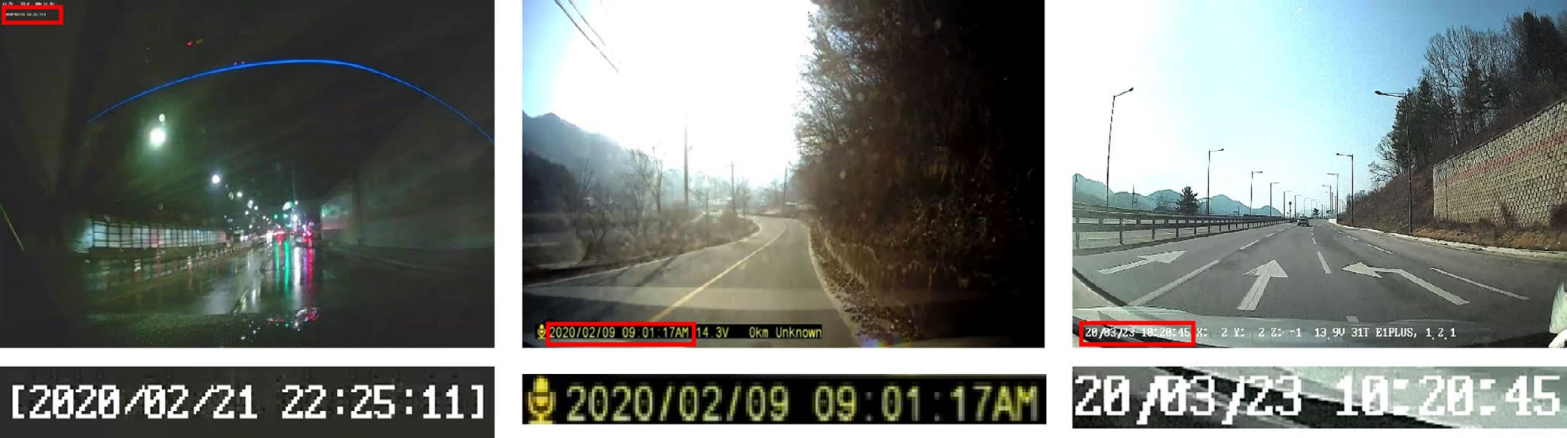
Displayed time information in dashcam videos [23].

To address this limitation, the methodology leverages the fact that video formats store supplementary information, such as GPS data, gyroscope sensor data, and timestamps, in separate tracks distinct from the primary video and audio streams. In the AVI format, as illustrated in Figure 8, timestamp information in various formats may be stored in dedicated metadata or subtitle chunks, such as “03tx,” “04tx,” “03st,” or “04st.” The most common timestamp implementation consists of a 4‐ or 6‐byte hexadecimal value. This value can be converted to its decimal equivalent, which typically represents elapsed time in seconds or milliseconds since the Unix epoch (January 1, 1970), thereby enabling precise determination of the video capture time.

**FIGURE 8:**
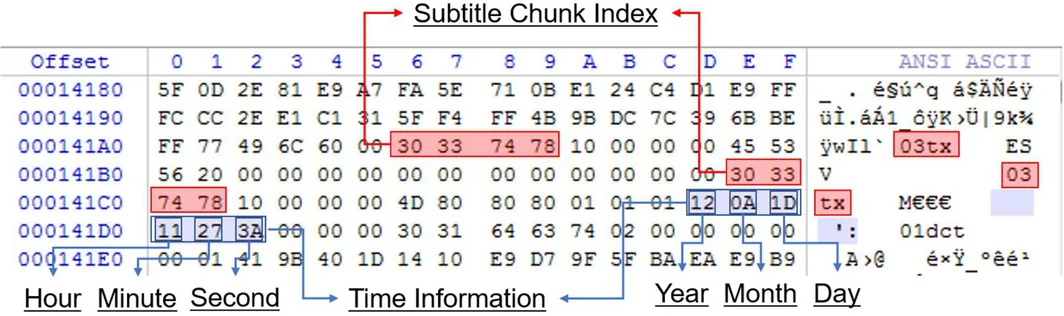
Subtitle chunk of the AVI container file format.

Similarly, in MP4 files, timestamp information may also be embedded between video frame data. As illustrated in Figure 9, some dashcam systems record the timestamp of a video frame in the NMEA GPRMC format. In this format, the second field represents the UTC time (hour, minute, and second), while the tenth field records the UTC date (day, month, and year) in text form. By parsing these fields, the exact recording time of the corresponding video frame can be determined.

**FIGURE 9:**
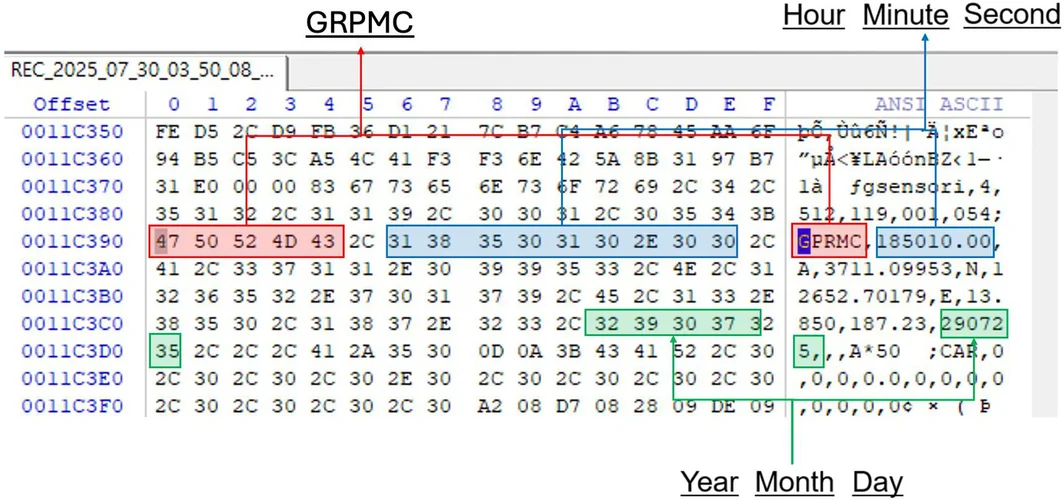
Time information in the MP4 container file format.

However, video fragments recovered from slack regions are typically small and discontinuous. Even when the original files were stored sequentially, the slack data extracted from each file is limited in size and does not preserve the original logical ordering of the video stream. This results in a highly fragmented distribution of video data with interspersed temporal metadata. Figure 10 illustrates this phenomenon by plotting the offset (*Y*‐axis) against the timestamp (*X*‐axis) of data fragments extracted from the dashboard camera memory card. As shown in the graph, older data exhibit a broad distribution of offsets, indicating severe fragmentation. In contrast, more recent data are clustered more contiguously, corresponding to a higher likelihood of recovering complete video segments.

**FIGURE 10:**
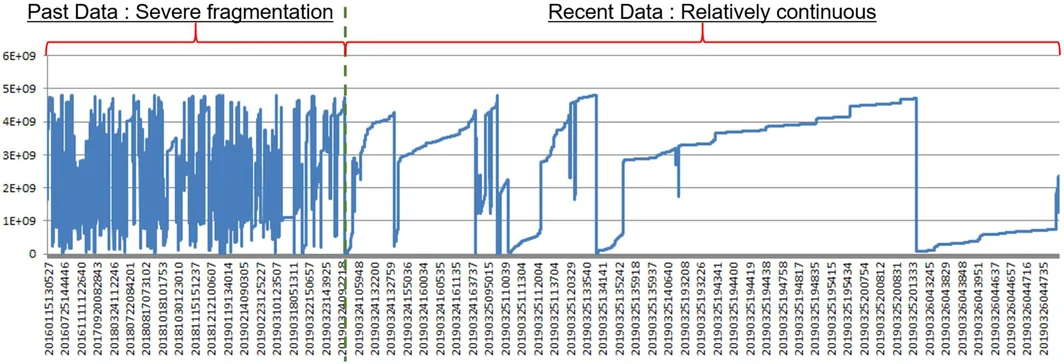
Correlation between extracted timestamps and slack offsets.

A naïve extraction of the video stream from this aggregated slack space that disregards embedded time information would inevitably result in a disjointed and chronologically incorrect sequence. This non‐sequential output substantially increases the risk of missing critical scenes related to an incident. The method proposed in this paper, therefore, implements a metadata‐driven reconstruction process. The algorithm first parses the extracted slack to locate timestamp metadata. It then reconstructs the H.264 stream data associated with the corresponding time information. Within this reconstructed stream, the method identifies IDR pictures (Intra‐frames), which serve as keyframe anchors. Once an IDR picture is identified, the algorithm searches for the connected group of non‐IDR pictures (Inter‐frames) that logically follow. Finally, these complete frame groups are sequentially connected and decoded to obtain a chronologically coherent and continuous video segment.

## 4 EXPERIMENT AND CASE STUDY

### 4.1 Experiment results

A series of experiments was conducted to evaluate the effectiveness of the proposed methodology. The experiments first identified and extracted video file slack from allocated files based on format‐specific structures using a specialized program developed for this purpose. This process aggregated all available video file slack data from each test dashboard camera storage medium. When the conventional recovery target, namely the unallocated area, is defined as ‘n’ bytes, the proposed method expands the total recoverable space to ‘n+’ α bytes. As demonstrated in Table 1, which compares pre‐allocating dashcam models, their corresponding file sizes, and the relative volumes of unallocated and slack areas in actual recovery cases, this expansion is substantial. The results indicate that the proposed approach increased the total data pool available for video recovery by a minimum of 26.7% and up to a maximum of 966.7%.

**TABLE 1:** Comparison of spaces to be recovered.

Case	Memory card capacity	Unallocated clusters (A)	File slack (B)	Rate of recovery space increase (%) (B/A*100)
INAVI V500 Thinkware	16 GB	420.4 MB	4064 MB	966.7%
GS3000FHD TAESUNG IT&D	16 GB	751 MB	854 MB	113.7%
INAVI QXD950View Thinkware	32 GB	728 MB	6714 MB	922.3%
ATOMGOLD‐S200 HYUNDAI MEIACE	16 GB	741 MB	438 MB	59.1%
INAVI V900 Thinkware	16 GB	411 MB	2046 MB	497.8%
INAVI Starview Mercedes‐Benz	32 GB	11,393 MB	3037 MB	26.7%

Beyond this quantitative expansion of the target area, the practical yield of forensically relevant artifacts was evaluated. Intra‐frames were extracted from both the unallocated area and the collected video file slack using codec information derived from existing allocated files as a reference. The results presented in Table 2 were informative. Certain dashcam models were identified that, despite having a substantial unallocated area, contained no recoverable video streams within it. In these critical scenarios, video file slack served as the sole source of recovered frames. Conversely, in test cases involving a recently formatted card with minimal subsequent use, the unallocated area was larger, as expected, and yielded a higher frame count. Importantly, even in these cases, forensically significant video streams were also recovered from the slack area, confirming it as a consistent and independent source of evidence.

**TABLE 2:** The number of recovered video frames (recovery rate).

Case	Unallocated clusters (A)	File slack (B)	Recovery rate (%) (B/A*100)
INAVI V500 Thinkware	0	2599	—
GS3000FHD TAESUNG IT&D	0	1384	—
INAVI QXD950View Thinkware	418	2618	626.3%
ATOMGOLD‐S200 HYUNDAI MEIACE	323	1120	346.7%
INAVI V900 Thinkware	0	1734	—
INAVI Starview Mercedes‐Benz^a^	9122	2347	25.7%

^a^ INAVI Starview (Mercedes‐Benz) dashboard cameras are automatically formatted every 21 days by their built‐in software, so they show relatively high proportion of unallocated space and many video frames recovered from the unallocated space.

A final critical finding emerged from correlating the timestamps extracted from the slack data with the timestamps of the currently allocated files, illustrated in Section 3. This analysis confirmed that all data recovered from the slack were temporally prior to the existing allocated data. These results demonstrate that the proposed method successfully recovers historical data from the overwritten portion of a pre‐allocated file, an area that is generally considered virtually unrecoverable using standard forensic tools. The complete recovery procedure proposed in this paper is summarized in the flowchart shown in Figure 11. In essence, the methodology complements existing forensic analyses. After completing a standard examination, the analyst examines the video file slack, collects the data, and then extracts and reconstructs it based on embedded time information to recover evidence that would otherwise be lost.

**FIGURE 11:**
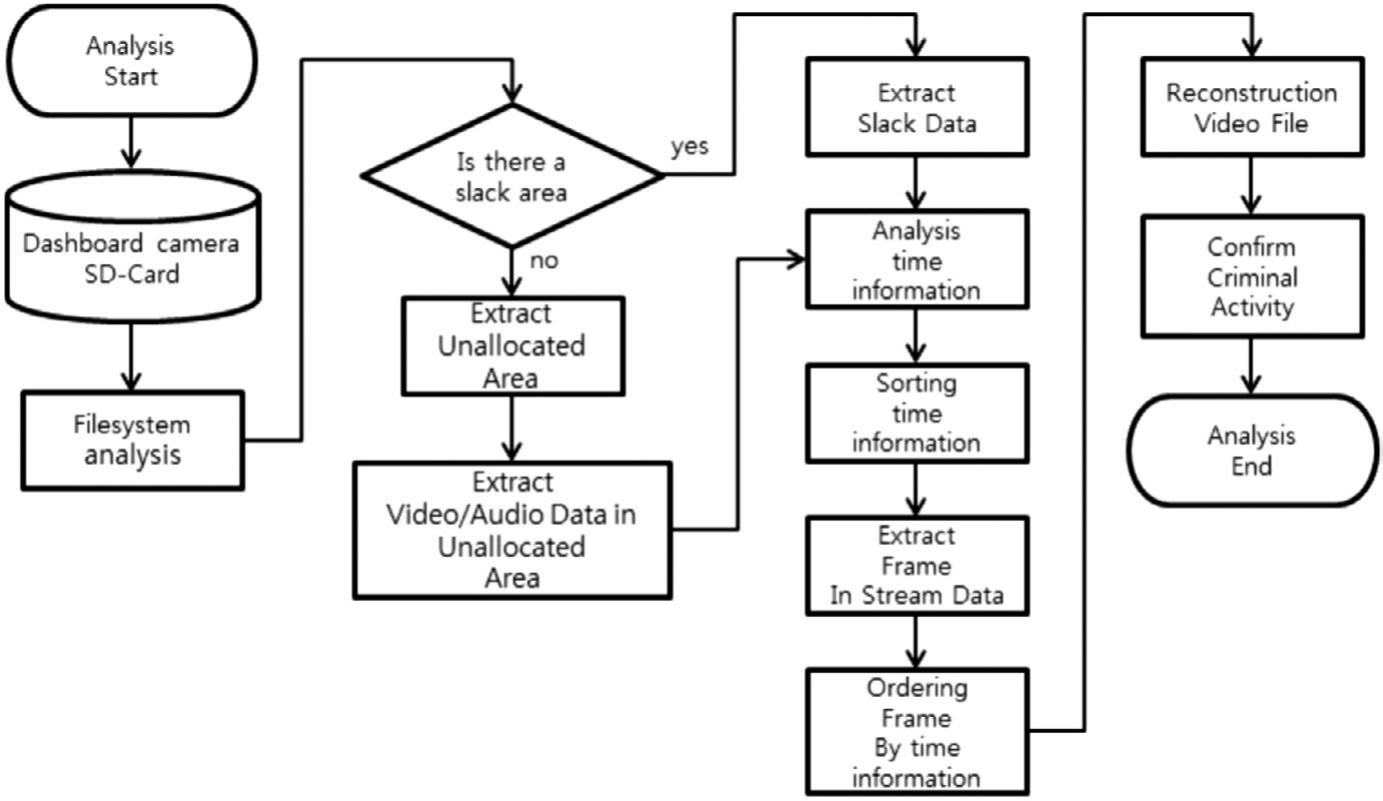
Dashboard camera memory card recovery process using the proposed method.

### 4.2 Case study: Recovery from a hit‐and‐run incident

To demonstrate the practical value and forensic applicability of the proposed methodology, it was applied to a real‐world criminal investigation. The case involved a suspected hit‐and‐run collision in which a driver, reportedly under the influence of alcohol, struck a pedestrian at night and fled the scene.

A significant challenge arose because the dashboard camera memory card was not secured by investigators until several days after the incident. Consequently, because of the device's continuous circular recording (loop recording) mechanism, the video files corresponding to the time of the accident had already been overwritten by newly recorded data. Standard file recovery methods targeting the unallocated area yielded no relevant video evidence.

However, by applying the proposed video file slack extraction technique to the currently allocated files on the storage medium, several partial video segments were successfully recovered, as shown in Figure 12. Analysis of these segments confirmed that they originated from the precise time of the incident. Critically, the recovered video frames contained clear visual evidence of the collision, directly capturing the moment of impact. This evidence, which would otherwise have been irretrievably lost, provided critical supporting evidence for the criminal charges.

**FIGURE 12:**
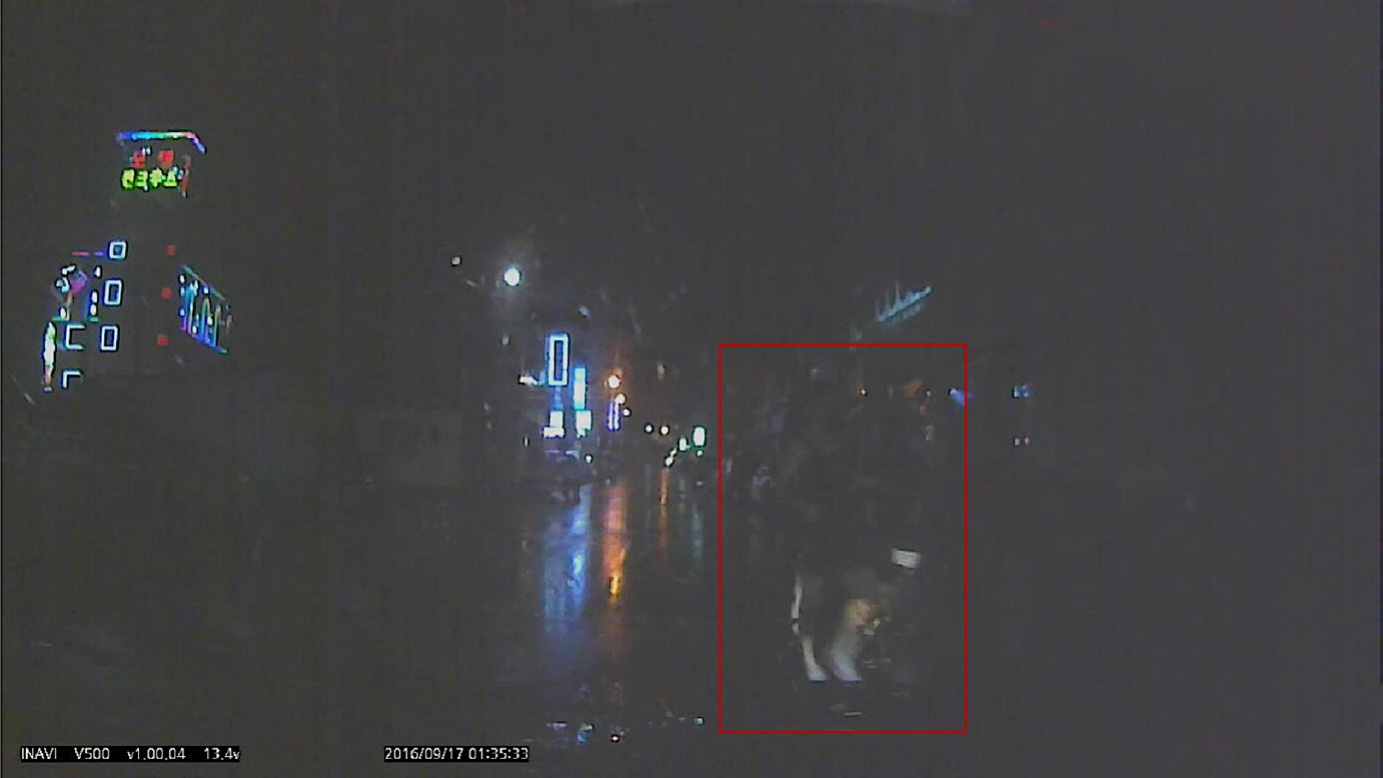
Scene of the accident recovered from the slack area.

These results suggest that, had the video file slack area, as defined in this study, not been specifically extracted and reconstructed, critical evidence for resolving the case would have been permanently overlooked. This emphasizes that the significance of this research lies not merely in addressing “unrecoverable” areas but in identifying and defining regions that were previously not even considered targets for recovery in standard forensic investigations. Ultimately, this methodology introduces a new analytical perspective for precisely securing residual data in loop‐recording devices such as dashboard cameras, thereby contributing to the maximization of practical forensic value.

## 5 CONCLUSIONS

This study identified and validated a novel and forensically significant evidence repository that has been largely overlooked, namely the “Video File Slack” generated by dashboard camera models that pre‐allocate fixed‐size files. This study formally defines the concept of “Video File Slack” as a new forensic artifact within pre‐allocated recording systems. The experimental findings demonstrate that for these prevalent device types, the recovery yield from this slack area can be substantial and, in some cases, comparable to or greater than that of the unallocated area. In several experiments, the number of recovered frames from the video file slack area significantly exceeded those obtained from traditional recovery targets.

A critical finding of this research is that in numerous test scenarios, particularly those simulating long‐term continuous use, the conventional recovery target, the unallocated area, was completely devoid of relevant video streams. In these scenarios, the proposed methodology successfully recovered a large volume of video frames from video file slack. This outcome indicates that traditional carving or recovery methods that focus solely on unallocated space are insufficient for comprehensive forensic examination of these devices. It should also be noted that the effectiveness of this approach depends on the recording architecture of the target device, particularly those employing fixed‐size pre‐allocation strategies.

Furthermore, analysis of embedded timestamp metadata within the recovered video frames empirically confirmed that the recovered data were chronologically prior to the oldest remaining allocated files. These results demonstrate that the technique retrieves historical data from storage regions that have already been logically overwritten and are generally considered unrecoverable using standard forensic tools. The implications for forensic practice are significant. In investigations where incident‐related video is presumed lost because of circular recording, the proposed method offers a viable pathway for recovering fragmented yet potentially crucial evidence. As demonstrated by the case study, even a small number of recovered frames can provide the critical information required to resolve a case.

The recovery of “past data” from overwritten data on a digital forensic target represents a meaningful and impactful endeavor. Based on these findings, video file slack should be considered in standard forensic examination procedures for dashcam forensics. Integrating slack‐based recovery techniques into existing forensic workflows would further enhance the completeness of digital evidence acquisition.

Accordingly, future work is clearly defined. There is a pressing need to develop an automated tool that institutionalizes this methodology. Such a tool should be capable of (1) analyzing a Dashcam memory card to determine whether it employs a pre‐allocation recording method, (2) automatically identifying and extracting video and audio streams from the resulting slack areas, and (3) reconstructing these fragmented streams into coherent and viewable video. Moreover, as this research demonstrates, the digital forensic field must continuously adapt. It is imperative to continue developing and refining forensic tools to address the increasing variety and complexity of data storage mechanisms used by dashboard camera manufacturers. Ultimately, this study demonstrates that previously overlooked storage artifacts can contain substantial evidentiary value when examined using context‐aware forensic techniques.

## FUNDING INFORMATION

This work was supported by National Forensic Service (NFS2025DTB01), Ministry of the Interior and Safety, Republic of Korea.

## CONFLICT OF INTEREST STATEMENT

The authors declare no conflicts of interest.

## CONSENT

All study participants provided informed consent.

## Data Availability

The data that support the findings of this study are available from the corresponding author upon reasonable request.
